# Condensin aids sister chromatid decatenation by topoisomerase II

**DOI:** 10.1093/nar/gkt882

**Published:** 2013-09-20

**Authors:** Adrian Charbin, Céline Bouchoux, Frank Uhlmann

**Affiliations:** Chromosome Segregation Laboratory, Cancer Research UK London Research Institute, London WC2A 3LY, UK

## Abstract

The condensin complex is a key determinant of mitotic chromosome architecture. In addition, condensin promotes resolution of sister chromatids during anaphase, a function that is conserved from prokaryotes to human. Anaphase bridges observed in cells lacking condensin are reminiscent of chromosome segregation failure after inactivation of topoisomerase II (topo II), the enzyme that removes catenanes persisting between sister chromatids following DNA replication. Circumstantial evidence has linked condensin to sister chromatid decatenation but, because of the difficulty of observing chromosome catenation, this link has remained indirect. Alternative models for how condensin facilitates chromosome resolution have been put forward. Here, we follow the catenation status of circular minichromosomes of three sizes during the *Saccharomyeces cerevisiae* cell cycle. Catenanes are produced during DNA replication and are for the most part swiftly resolved during and following S-phase, aided by sister chromatid separation. Complete resolution, however, requires the condensin complex, a dependency that becomes more pronounced with increasing chromosome size. Our results provide evidence that condensin prevents deleterious anaphase bridges during chromosome segregation by promoting sister chromatid decatenation.

## INTRODUCTION

The chromosomal condensin complex is best known for its role in promoting mitotic chromosome condensation. In addition, it is required for sister chromatid resolution during their segregation in anaphase (1–4). The two activities of condensin are separable. Inactivation of budding yeast condensin results in 1.5-fold longer chromosome arms in mitosis, which should prevent only the longest chromosomes from fully segregating, but the resolution of most chromosomes is impaired (3,5,6). Furthermore, Aurora B kinase is required for condensin-dependent compaction, but not resolution, of the budding yeast ribosomal DNA (rDNA) locus (7,8). A role of condensin in both chromosome compaction and resolution is conserved in higher eukaryotes (4,9–13). Similarly, the condensin-related SMC complex in prokaryotes is required for both compaction and segregation of the bacterial nucleoid, implying that chromosome resolution is a fundamental aspect of condensin function (14,15).

The chromosome segregation failure in condensin mutant yeast cells is reminiscent of the phenotype observed after inactivation of topoisomerase II (topo II), an enzyme that removes catenanes that persist between sister chromatids following DNA replication (16–18). It has therefore been suggested that condensin facilitates DNA decatenation by topoisomerase II. Stimulation of topo II by condensin was reported in *Drosophila*, but could not be seen in *Xenopus* or yeast (4,5,19). Alternative models in which condensin resolves transcription-dependent chromosome links have therefore been put forward (20,21). Recent studies in prokaryotes have reported a direct protein interaction between the *Escherichia coli* condensin subunit MukB and the *E. coli* decatenating topoisomerase topo IV (22,23). However, the impact of this interaction on the decatenation activity of topo IV remained weak and an alternative role of the MukB–topo IV interaction in chromosome folding has been suggested (24). Whether and how chromosome folding might indirectly facilitate sister chromatid decatenation remains an interesting open question.

A reason why the interplay between condensin and topo II in chromosome resolution remains poorly understood is the difficulty of visualizing sister chromatid catenation. Intertwinings between linear chromosomes are not maintained after DNA isolation and cannot therefore be visualized by conventional gel electrophoresis. Catenanes between circular chromosomes, however, can be visualized. This has early on been used to characterize the cell-cycle dependence of catenane formation in budding yeast (25) with an aim of investigating whether catenanes contribute to sister chromatid cohesion. This latter question has recently been addressed in more detail and a role for cohesin, but not condensin, in maintaining catenanes after their synthesis in S-phase has been documented (26). These studies have not addressed how catenanes that persist between sister chromatids following DNA replication are eventually resolved. This was the topic of another recent study that correlated condensin and microtubule-dependent positive supercoiling of plasmid DNA *in vivo* with its facilitated decatenation by topo II *in vitro* (27). A possible role of condensin in DNA decatenation *in vivo* was not addressed in this study due to the difficulty of visualizing plasmid catenanes.

Despite the ubiquitous occurrence of DNA catenation as the consequence of DNA replication, and the importance of its resolution, chromosome decatenation during mitosis has not yet been directly observed *in vivo*. Here, we follow the catenation status of circular minichromosomes of three sizes during cell-cycle progression in budding yeast. We find that the majority of catenanes produced during DNA replication are rapidly resolved, but that complete catenane removal requires condensin, a dependency that becomes more pronounced as chromosome size increases. These results provide evidence that condensin promotes sister chromatid decatenation to facilitate eukaryotic chromosome segregation.

## MATERIALS AND METHODS

### Yeast strains and culture

The strains used in this study were of w303 background, with the exception of the *brn1-9* and *smc4-1* strains that were of S288c background. Detailed genotypes are found in Supplementary Table S1. The centromeric plasmid pRS316 and ring chromosome RCIII were as described (28,29). pS14-8 contains a genomic region surrounding *RAD5* in plasmid YCp70 and was a gift from A.-M. Farcas and K. Nasmyth. pRS316 and pS14-8 were detected by Southern blotting using a probe against the *amp^R^* gene. RCIII was detected with a probe against *LEU2*, while the *leu2-3.112* gene on the authentic chromosome III was replaced with a *kan^R^* marker. Anchor-away strains were created as described (30). To deplete nuclei of cohesin or condensin, rapamycin was added to the anchor-away strains at the time of G1 release. Cells of the **a** mating type were synchronized in G1 using 0.5 µg/ml α-factor, while cells of **α** mating type were synchronized using 0.04 µg/ml **a**-factor, as described (31). After release, pheromone was added back to the culture after all cells budded for rearrest in the following G1. Arrest in metaphase was achieved by Cdc20 depletion (32), or by addition of 5 µg/ml nocodazole to the culture. FACS analysis of DNA content and immunofluorescence microscopy followed standard procedures.

### Minichromosome purification, electrophoresis and quantification

One gram of cells were collected at each time point and resuspended in ice cold 1 M sorbitol, 0.1 M EDTA pH 7.5, 0.02% sodium azide. Cell pellets were then resuspended in 0.5 ml of the same buffer, 20 µl of 2.5 mg/ml Zymolyase 100T (MP Biomedicals) was added and the suspension incubated for 1 h at 37°C with mild agitation. Cells were collected by centrifugation for 10 min at 13 000 rpm, supernatants removed by aspiration and the pellets resuspended in 0.5 ml of 50 mM Tris–HCl pH 7.4, 20 mM EDTA. SDS was added to a final concentration of 1% and samples were incubated at 65°C for 15 min. An amount of 0.2 ml of 5 M potassium acetate was added and the samples placed on ice for 1 h. The precipitate was removed by centrifugation for 5 min at 14 800 rpm at 4°C and the supernatants collected. The centrifugation step was repeated until the supernatant fractions were clear of any debris. Two volumes of 100% ethanol were added and after 5 min the DNA was collected by centrifugation for 1 min at 13 000 rpm. The supernatants were aspirated and the pellets allowed to air dry. Once dry, pellets were carefully resuspended in 0.3 ml of TE, pH 7.4 and 50 µg/ml RNaseA added and incubated for 30 min at 37°C, before addition of 100 µg/ml proteinase K for a further 30 min. Now, 12.6 µl of 5 M sodium chloride was added, and the DNA precipitated by addition of 0.63 ml 100% ethanol. Samples were again centrifuged, supernatants aspirated and the pellets air dried. After taking up in TE, the DNA concentration was determined and adjusted prior to gel electrophoresis.

Samples were resolved on 0.5% agarose/TAE gels at 1.0 V/cm for 48 h (pRS316 and pS14-8) at room temperature, or at 0.8 V/cm for 24 h, followed by 2.2 V/cm for a further 24 h (RCIII). Southern transfer was carried out using capillary blotting onto a positively charged nylon membrane (Hybond-N^+^, GE Healthcare). The membrane was probed with random-prime ^32^P-labeled probes. The blots were exposed to PhosphorImager screens that were scanned using a Storm 860 Molecular Imager (GE Healthcare). Band intensities were analyzed and quantified using automatic band detection and linear background subtraction in ImageQuant.

Enzymes used to verify the nature of the observed bands were XhoI, PmlI and Nt.BstNBI (New England Biolabs), calf thymus topo I (Invitrogen), human recombinant topo I and human topo IIα (both Topogen).

## RESULTS

### Catenane formation and resolution during the cell cycle

**Figure 1.**
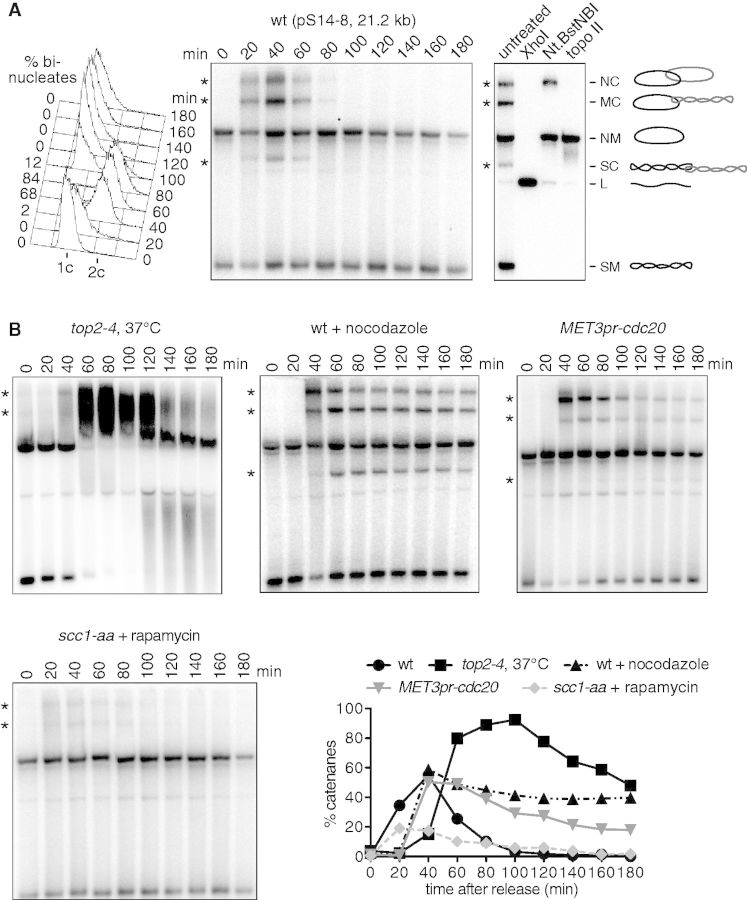
Catenane formation during DNA replication and their resolution. (**A**) A wild-type strain carrying minichromosome pS14-8 was synchronized in G1 by α-factor treatment and samples taken at 20-min intervals after release. α-factor was added back to the culture for re-arrest in the next G1. FACS analysis of DNA content, the percentage of binucleate cells and the Southern blot to visualize pS14-8 are shown. Enzyme digests of the 40-min time point, shown on the right, allow band identification as indicated. Nt.BstNBI is a nicking endonuclease. NC, nicked catenanes; MC, mixed catenanes; NM, nicked monomers; SC, supercoiled catenanes; L, linear form; SM, supercoiled monomers. Catenated species are highlighted by asterisks (*). (**B**) as (**A**), but the indicated strains were used. FACS analysis of DNA content as a marker of cell-cycle progression for each culture is contained in Supplementary Figure S1. The *top2-4* strain was released from α-factor arrest at 37°C, a wild-type control at that temperature is shown in Supplementary Figure S3. Each band species on the Southern blots was quantified and the percentage catenanes is plotted over time.

To investigate the formation and resolution of sister chromatid catenation during the budding yeast cell cycle, we visualized a 21.2-kb circular minichromosome (pS14-8) containing a section of chromosome XII as well as the centromere and replication origin (*ARS1*) of chromosome IV. First, wild-type cells were arrested in G1 and released into a synchronous cell cycle. Samples were collected every 20 min and DNA was extracted for Southern blotting. A probe specific for pS14-8 revealed a changing pattern of topological isoforms with different electrophoretic mobility (Figure 1A). To determine the identity of the different bands, we treated a sample from S-phase (40 min after release) with various enzymes. Restriction digest of the plasmid using XhoI collapsed all isoforms into a single linear band (L). A faint band visible throughout the timecourse at this position is probably an artifact due to DNA shearing during DNA purification. Incubation with the nicking endonuclease Nt.BstNBI relaxes supercoiled isoforms and resolved all species into two bands corresponding to nicked open circular species. These two bands were subsequently identified as representing nicked monomer (NM) and nicked catenated (NC) species after incubation with topo II, which resolved the catenanes to generate monomer species. Treatment with topo II also generated a faint smear below the monomer band that likely corresponds to partially supercoiled monomers. The band identity based on these enzyme treatments is indicated in Figure 1A, supporting the notion that catenated forms of pS14-8 arise during DNA replication and are resolved as the cell-cycle progresses.

When we quantified the fraction of catenated species during the time course, we found that it never reached more than about half of the total (Figure 1B). This was surprising as the efficient *ARS1* replication origin is expected to fire in every cell cycle. If catenation arises as the consequence of DNA replication, we would expect all of the minichromosomes to become at least transiently catenated. It could therefore be that DNA replication does not always lead to catenation between replication products. Alternatively, some catenanes might be resolved soon after DNA synthesis. To distinguish between these two possibilities, we repeated the time course analysis but inactivated topo II, the main cellular decatenase, using the temperature sensitive *top2-4* mutation (33). Now, virtually all of the minichromosomes accumulated as catenanes following DNA replication (Figure 1B). Their electrophoretic mobility was less well defined, probably because their supercoiling status following DNA replication was also affected by topo II inactivation. However, conversion of the catenanes to monomers by *in vitro* topo II treatment confirmed their identity (Supplementary Figure S1). At later time points, concomitantly with anaphase onset, the appearance of a smear below the linear form is consistent with the possibility that the minichromosome broke as a result of chromosome segregation. Taken together, this suggests that all replication products arise as catenanes, but that in wild-type cells a substantial part of these is resolved by topo II soon after their synthesis.

To estimate the fraction of catenanes that persist after DNA replication, wild-type cells were released from G1 into a mitotic arrest using either the spindle poison nocodazole or by depletion of the anaphase promoting complex coactivator Cdc20 (Figure 1B). In the presence of nocodazole, after a peak of catenane formation during S-phase, ∼40% of the minichromosomes remained catenated in mitosis. When we used Cdc20 depletion to arrest cells in mitosis, only ∼20% of catenated molecules persisted. This observation suggests that the pulling force of the mitotic spindle, that is present after Cdc20 depletion but is destroyed by nocodazole, aids sister chromatid decatenation. We cannot formally exclude that the mitotic checkpoint, activated in nocodazole-treated cells, impacts on chromatid decatenation. Support for the idea that spindle-dependent chromosome movement aids decatenation comes from an additional experiment in which we depleted cohesin, the chromosomal protein complex that restricts sister chromatid separation. When we depleted nuclear cohesin using an anchor-away allele of its Scc1 subunit (*scc1-aa*) (30, Supplementary Figure S2), the abundance of catenanes during the cell cycle was markedly reduced. Even at the peak during S-phase, <20% of catenanes were detected. Consistent with a previous report (26), this suggests that a substantial fraction of catenanes are readily resolved by topo II as sister chromatids move apart.

### Condensin is required to complete minichromosome decatenation

**Figure 2.**
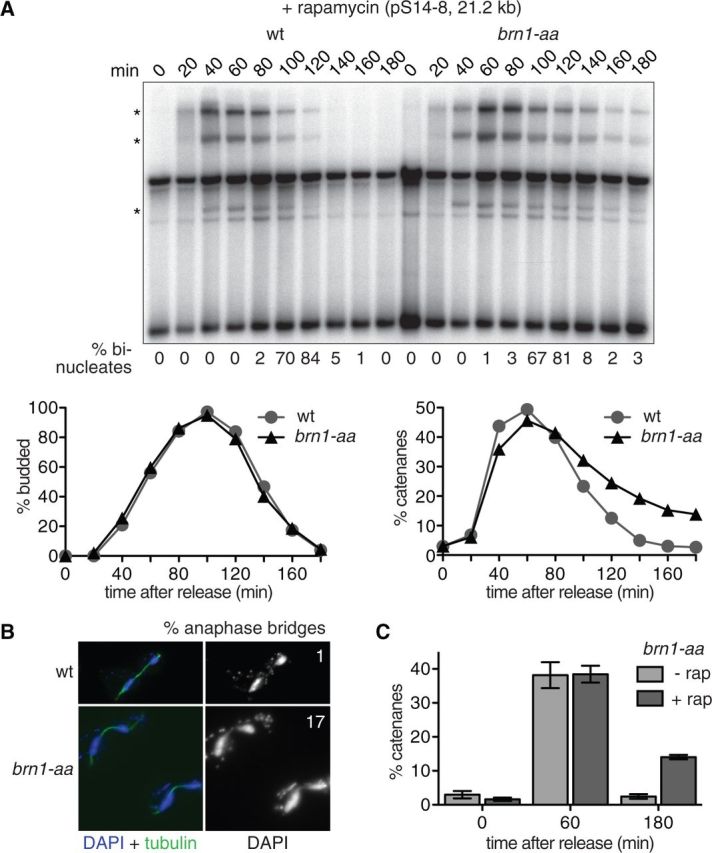
Condensin promotes minichromosome decatenation. (**A**) Condensin was depleted by rapamycin-induced anchor-away of its Brn1 subunit in the *brn1-aa* cells, at the time of release from an **a**-factor induced G1 arrest. **a**-factor was added back to the culture for re-arrest in the next G1. The budding index and the percentage of binucleated cells are shown as markers of cell-cycle progression, as well as the Southern blot to visualize the pS14-8 minichromosome and the quantification of the catenanes, highlighted by asterisks (*). (**B**) Immunofluorescence microscopy of cells stained with an α-tubulin antibody and the DNA dye 4′,6-diamidino-2-phenylindole (DAPI) reveals anaphase bridges after condensin anchor-away. The frequency of these bridges in late anaphase cells with fully elongated spindles, at 140 min, is indicated (*n* = 100). (**C**) An experiment as shown in (**A**) was repeated in triplicate in the *brn1-aa* strain in the absence or presence of rapamycin. The percentage of catenanes at the indicated time points was quantified. The means and standard error are shown.

To study the contribution of condensin to sister chromatid decatenation during the cell cycle, we depleted nuclear condensin using anchor-away of its Brn1 subunit (*brn1-aa*) (30). Wild-type cells, or cells carrying the *brn1-aa* allele, were released from G1 into a synchronous cell cycle in presence of rapamycin. Catenanes accumulated during S-phase to similar levels, however, catenane resolution progressed more slowly in condensin-depleted cells and ∼15% catenanes persisted in the *brn1-aa* cells after they had returned to G1 at the end of the experiment (Figure 2A). Condensin depletion was accompanied by characteristic chromosome bridges in late anaphase, a hallmark of chromosome missegregation following condensin inactivation (Figure 2B). A similar condensin requirement for complete minichromosome decatenation was reproducibly observed in several replicates of an experiment in which we compared *brn1-aa* cells progressing through the cell cycle with or without rapamycin addition (Figure 2C). We also observed persistent catenanes following condensin inactivation using the *brn1-9*, *smc4-1* and *ycg1-10* thermosensitive alleles in two different *Saccharomyces cerevisiae* strain backgrounds (Supplementary Figure S3). This demonstrates that condensin is required for completion of sister chromatid decatenation.

Persistent catenanes in the absence of condensin offer an explanation for anaphase bridges and chromosome missegregation. One caveat to this conclusion is that if condensin was required to resolve catenation-independent linkages on authentic chromosomes, then the resulting nuclear segregation defect might indirectly hamper minichromosome decatenation. We therefore examined whether condensin impacts on decatenation independently of chromosome movement. To do this, we arrested cells in mitosis using nocodazole, a situation where chromosome movement no longer contributes to sister chromatid resolution. Cohesin was depleted using the *scc1-aa* allele to allow spontaneous catenane resolution. Co-depletion of condensin led to a small but reproducible increase of persisting catenanes (Supplementary Figure S4A). This suggests that condensin promotes chromosome decatenation independently of chromosome movement in anaphase.

As additional control, we used cells arrested in mitosis by depletion of Cdc20 when cohesin persists. Sister chromatid decatenation under these conditions was again reproducibly facilitated by condensin (Supplementary Figure S4B). Together this confirms that condensin facilitates chromosome decatenation independently of cohesin cleavage and of chromosome segregation.

### Condensin’s decatenation role increases with chromosome size

**Figure 3.**
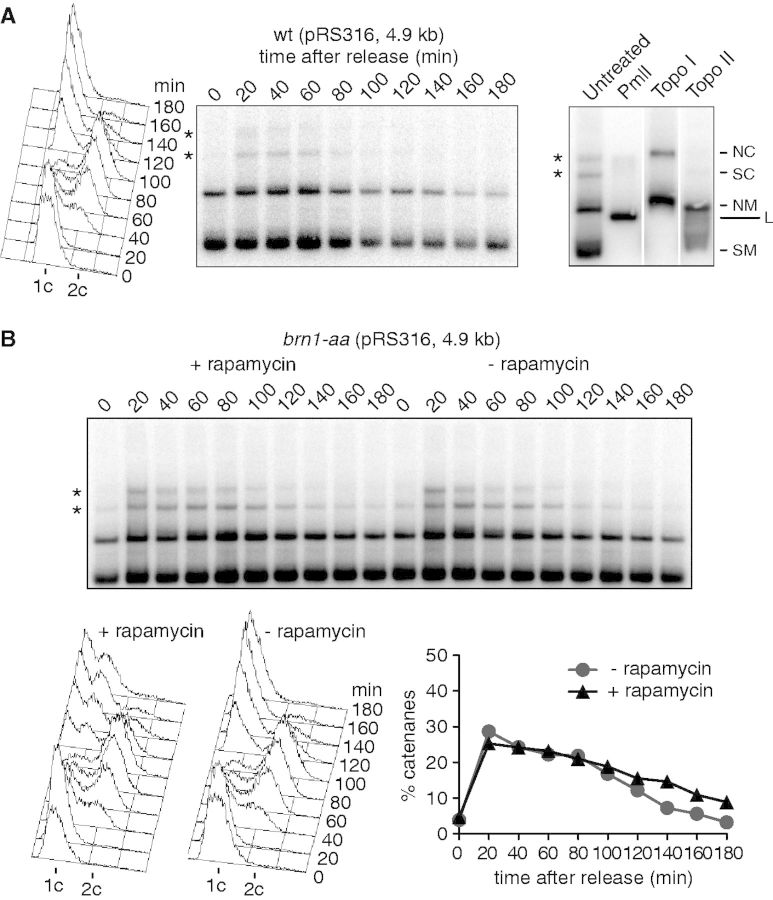
A less pronounced condensin requirement for small plasmid decatenation. **(A**) The catenation status of the centromeric plasmid pRS316 was analyzed during synchronous cell-cycle progression. FACS analysis of DNA content and the Southern blot to visualize pRS316 are shown. Enzyme digests of the 40 min time point allow band identification as indicated. Abbreviations are as in Figure 1, catenated species are highlighted by asterisks (*). (**B**) The pRS316 catenation status was analyzed during synchronous cell-cycle progression in the presence or absence of rapamycin to anchor-away condensin using the *brn1-aa* allele. FACS analysis of DNA content is shown, as well as the Southern blot to visualize pRS316 and the quantification of catenanes.

We next investigated how chromosome size affects the condensin requirement for decatenation. When using a small 4.9-kb plasmid (pRS316), we again detected accumulation of catenanes during S-phase, that were subsequently resolved (Figure 3A). pRS316 catenanes were less abundant, compared to the pS14-8 minichromosome, suggesting that the majority of the plasmid is rapidly decatenated after its replication. Nuclear depletion of condensin using the *brn1-aa* allele caused only a small delay to plasmid resolution (Figure 3B), even though overall chromosome segregation was again strongly impeded, as seen by FACS analysis of the cellular DNA content. This suggests that the condensin requirement for resolution of a small plasmid is less pronounced than in the case of a larger minichromosome.

**Figure 4.**
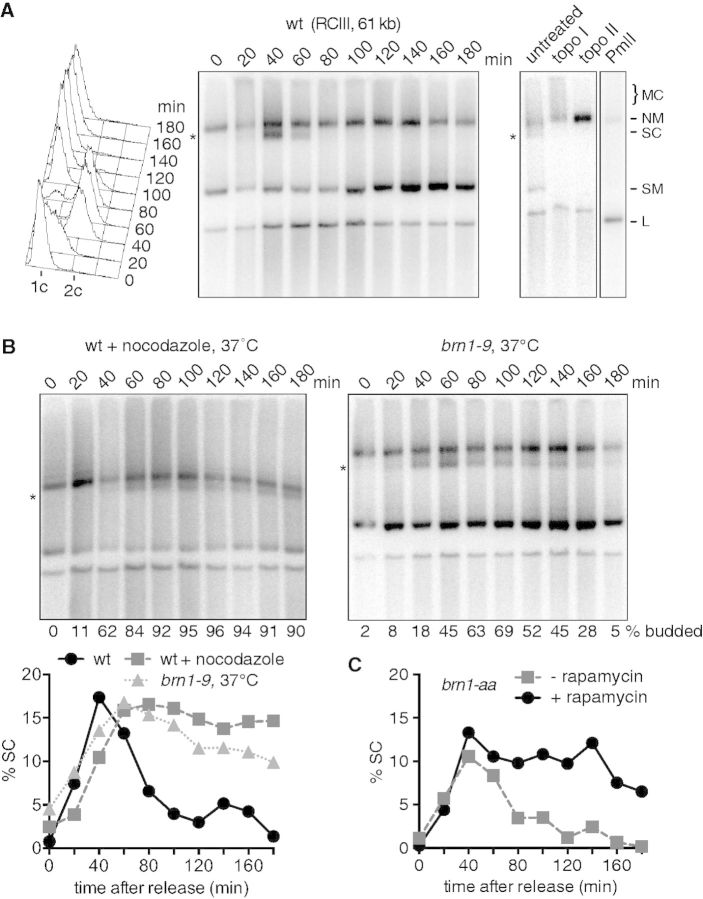
A marked role for condensin in ring chromosome III (RCIII) decatenation. (**A**) The RCIII catenation status was analyzed in synchronized wild-type cells. FACS analysis of DNA content is shown, as well as the Southern blot to visualize RCIII together with enzyme digests that allow the indicated band assignments. Abbreviations are as in Figure 1. Note that the diagnostic band used for quantification, highlighted by an asterisk (*), represents only the supercoiled catenanes (SC) and therefore only a subset of all catenated forms. (**B**) Catenanes persist in a mitotic arrest or if condensin is inactivated by the *brn1-9* allele. As in (**A**), but cells were released from G1 into nocodazole-containing medium or at 37°C to inactivate the *brn1-9* allele. The fraction of supercoiled catenanes (SC) was quantified and is plotted as a function of time. (**C**) RCIII catenation and decatenation was assessed in a synchronous culture of *brn1-aa* cells in the absence or presence of rapamycin to anchor-away condensin. The FACS analysis of DNA content, as an indicator of cell-cycle progression, is shown in Supplementary Figure S1. The percentage of supercoiled catenanes is plotted as a function of time.

We next studied a 61-kb long ring chromosome (RCIII), derived from yeast chromosome III, including its centromere and three replication origins (29). Separation of its topological isoforms by gel electrophoresis was more demanding and a greater background made band identification and quantification difficult. Despite these drawbacks, a discrete band appeared below the nicked monomer (NM) band during DNA replication that we identified by enzyme treatments as supercoiled catenanes (SC) (Figure 4A). Only part of the ring chromosome appeared catenated following S-phase, suggesting that a sizeable portion even of this large substrate is decatenated rapidly. When we blocked mitotic progression using nocodazole, a similar level of the diagnostic SC band became detectable during S-phase, but these catenanes were no longer resolved and persisted for an extended period in the arrest (Figure 4B). We then inactivated condensin using the *brn1-9* allele. In this case, cells progressed through mitosis, yet catenane resolution was inefficient and the majority of catenanes persisted throughout the remainder of the time course when cells had returned to G1. The same was observed after condensin anchor-away using the *brn1-aa* allele (Figure 4C).

Our results with circular chromosomes of three sizes confirm that condensin is required for complete sister chromatid decatenation. It appears that two pools of catenanes are formed in S-phase. A substantial fraction of catenanes is readily and swiftly resolved following DNA synthesis, while a second pool of catenanes persists that require condensin for their resolution. The condensin requirement for resolution of this latter pool was greatest in case of the 61-kb ring chromosome, while it was less pronounced for the 21.2-kb minichromosome and barely detectable in case of the 4.9-kb plasmid.

### The interplay between condensin and topo II

Condensin and topo II show an overlapping localization pattern along budding yeast chromosomes (6), though it has remained controversial whether condensin directly interacts with topo II. Enzymatic stimulation of *Drosophila* topo II by condensin has been reported (4,34), but these results could not be reproduced by others in *Xenopus* (19). Recently, a physical interaction between the *E. coli* condensin and topo II orthologs has been reported, the consequences of which are still being explored (22–24). To shed insight into the interplay between condensin and topo II, we purified both budding yeast enzymes. Even though each demonstrated its expected biochemical activities, condensin did not stimulate the decatenation activity of topo II *in vitro* (Supplementary Figure S5). We conclude that condensin promotes sister chromatid decatenation in a manner that is distinct from activation of topo II’s catalytic activity.

## DISCUSSION

A large fraction of catenanes that are produced during DNA replication, at least between circular chromosomes, are readily and rapidly resolved by topo II. However, our results show that a subset of catenanes persists for extended periods and require condensin for their resolution. Is there a distinction between the two types of catenanes? One possibility is that all replicated chromosomes are left catenated in the same way, possibly by a small number of interlinks per replication termination event. Condensin could promote their resolution by instructing a substrate geometry that facilitates recognition by topo II (35). It has been suggested that under the force of the mitotic spindle condensin promotes a change of negative to positive supercoiling, at least of a small yeast plasmid. Positively supercoiled substrates in turn appear to be preferred topo II substrates (24,27). Whether condensin can instruct and maintain a force-dependent change in supercoiling along large eukaryotic chromosomes arms to facilitate their decatenation remains to be explored. In an alternative scenario, different types of topological interlinks between sister chromatids might exist. Simple catenanes might be good substrates for rapid decatenation by topo II, while condensin would be required to aid the resolution of more complex topologies, e.g. knots (36). Such complex structures might form less frequently during replication termination but at the same time might pose the greatest threat to chromosome segregation.

Anaphase bridges, like those seen in cells with compromised condensin function, are thought to be a major cause of chromosome instability in mammalian cells (37). An important area for future research is to investigate whether resolution of catenation between linear chromosomes in yeast and higher eukaryotes similarly depends on condensin. The catenation status of linear chromosomes cannot be assessed using conventional gel electrophoretic techniques, so this will require the development of new experimental tools. The danger of persisting anaphase bridges to genome stability makes this an important area of further study.

## SUPPLEMENTARY DATA

Supplementary Data are available at NAR online [38–41].

## FUNDING

The European Research Council [grant number ERC-2009-AdG-20090506]; Cancer Research UK and by a Boehringer Ingelheim Fonds PhD Fellowship [to A. C.]. Funding for open access charge: Cancer Research UK.

*Conflict of interest statement*. None declared.

## Supplementary Material

Supplementary Data
