# Supplementary material for: Condensin aids sister chromatid decatenation by topoisomerase II
Source: Nucleic Acids Res. 2013 Sep 20;42(1):340–8. doi: 10.1093/nar/gkt882 (PMC3874195; doi:10.1093/nar/gkt882)
Supplement: Supplementary Data [file supp_42_1_340__index.html]

Condensin aids sister chromatid decatenation by topoisomerase II — Supplementary Data 

# Condensin aids sister chromatid decatenation by topoisomerase II

## Supplementary Data

files

**Files in this Data Supplement:**

- Supplementary Data - pdf file
