# Supplementary material for: Condensin aids sister chromatid decatenation by topoisomerase II
Source: Nucleic Acids Res. 2013 Sep 20;42(1):340–8. doi: 10.1093/nar/gkt882 (PMC3874195; doi:10.1093/nar/gkt882)
Supplement: Supplementary Data [file supp_gkt882_nar-00791-h-2013-File006.pdf]

Supplementary Table S1. Yeast strains used in this study.

Figure 1

Y2665	*MAT***a***, ade2-1, trp1-1, can1-100, KAN, his3-11, ura3-52, GAL, psi+,* (w303 wild type), containing *pS14-8(LEU2)*
Y4007	*MAT***a***, top2-4, pS14-8(LEU2)*
Y4129	*MAT***a***, MET3pr-HA-CDC20::URA3, pS14-8(LEU2)*
Y4201	*MATα tor1-1, fpr1::NAT, RPL13A-2xFKB12::TRP1, scc1-FRB::HIS3, pS14-8(LEU2)*

Figure 2

Y4059	*MATα brn1-FRB::HIS3, tor1-1, fpr1::NAT, RPL13A-2xFKB12::TRP1, pS14-8(LEU2)*
Y4332	*MATα tor1-1, fpr1::NAT, RPL13A-2xFKB12::TRP1, pS14-8(LEU2)*

Figure 3

Y4199	*MAT***a***, pRS316(URA3)*
Y4333	*MATα tor1-1, fpr1::NAT, RPL13A-2xFKB12::TRP1, brn1-FRB::HIS3, pRS316(URA3)*

Figure 4

Y4259	*MAT***a***, leu2Δ::kan*^{R}*, RCIII-SUP11-LEU2-3ARS*
Y4264	*MAT***a***, leu2Δ::kan*^{R}*, brn1-9::TRP1, RCIII-SUP11-LEU2-3ARS*
Y4327	*MAT***a***, leu2Δ::kan*^{R}*, brn1-FRB::HIS3, tor1-1, fpr1::NAT, RPL13A-2xFKB12::TRP1, RCIII-SUP11-LEU2-3ARS*

Supplementary Figure S1, strains from Figures 1 and 4

Supplementary Figure S2

Y4235	*MATα tor1-1, fpr1::NAT, RPL13A-2xFKB12::TRP1, scc1-FRB::HIS3, smc2-FRB::kan*^{R}*, pS14-8(LEU2)*

Supplementary Figure S3

Y2665	as Figure 1
Y3940	*MAT***a***, NET1-GFP::TRP1, ycg1-10, pS14-8(LEU2)*
Y3939	*MAT***a***, brn1-9, pS14-8(LEU2)* (S288c background)
Y3954	*MAT***a***, smc4-1, pS14-8(LEU2)* (S288c background)

Supplementary Figure S4

Y4201	as Figure 1
Y4235	*MATα tor1-1, fpr1::NAT, RPL13A-2xFKB12::TRP1, scc1-FRB::HIS3, smc2-FRB::kan^{R}, pS14-8(LEU2)*
Y4058	*MATα tor1-1, fpr1::NAT, RPL13A-2xFKB12::TRP1, smc2-FRB::HIS3, pS14-8(LEU2)*
Y4113	*MATα MET3pr-HA-CDC20::URA3, tor1-1, fpr1::NAT, RPL13A-2xFKB12::TRP1, smc2-FRB::HIS3, pS14-8(LEU2)*

Supplementary Figure S5

D1090	*MAT**a**, lys2::PGAL1-GAL4, pep4::HIS3, bar1::hisG, p490(2m URA3 leu2-d GAL-SMC4-SMC2-BRN1), p491 (2μ TRP1 leu2-d GAL-YCS4-YCG1)*
JEL1	*MAT**a**, top1^{-}, YEpTOP2-PGAL1*

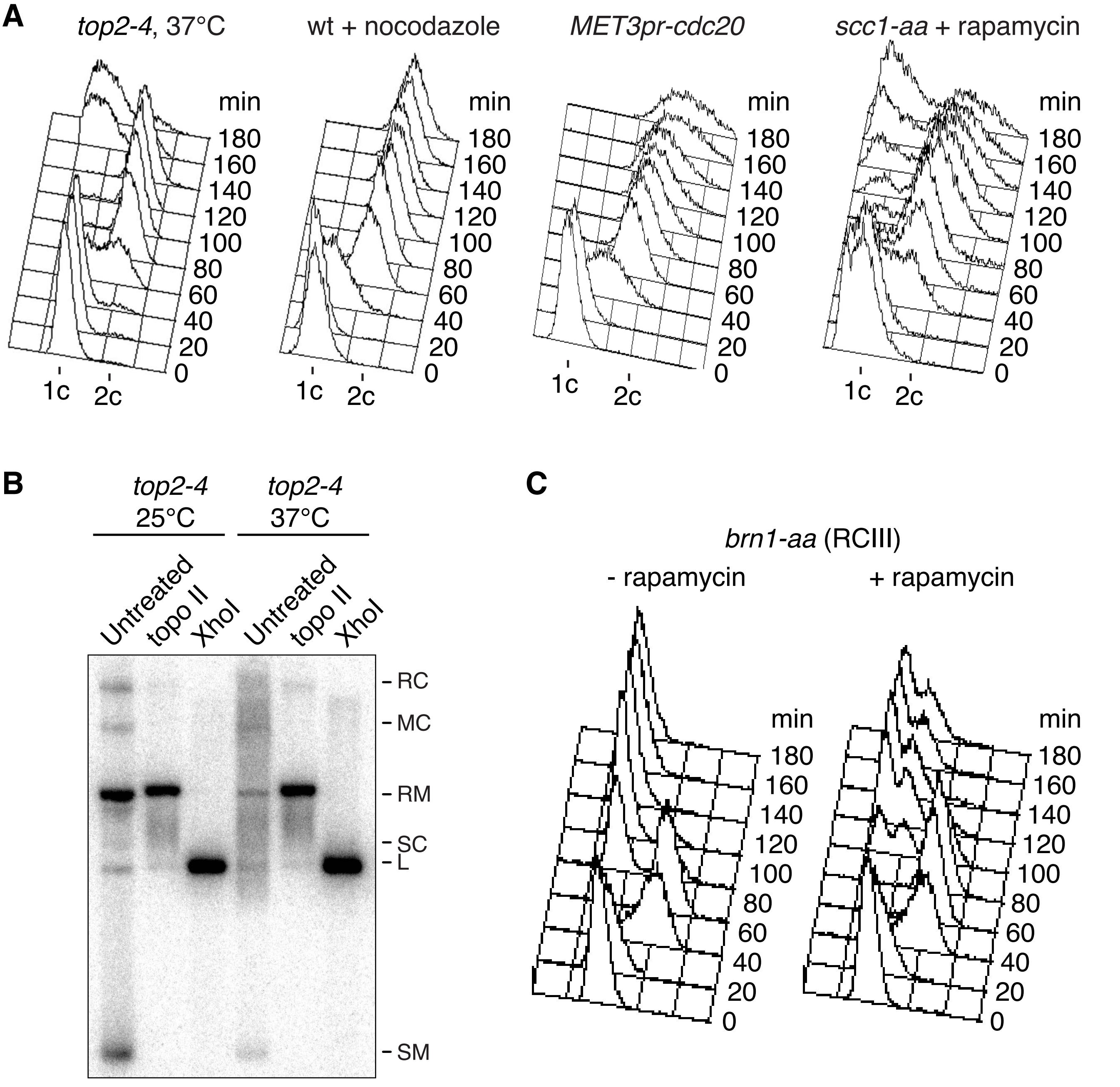

Supplementary Figure S1. Additional controls supporting conclusions from Figure 1 and Figure 4. (**A**) FACS analysis of DNA content to monitor cell cycle progression during the experiments shown in Figure 1B. (**B**) The minichromosome pS14-8 accumulates in catenated isoforms in a *top2-4* strain at restrictive temperature. An S-phase DNA sample from a *top2-4* strain, progressing through the cell cycle either at permissive (25°C) or restrictive (37°C) temperature, was *in vitro* treated with purified topo II or with the restriction enzyme XhoI. Most of the isoforms in both conditions are resolved by topo II into relaxed monomers. A smear below the relaxed monomers is likely due to partially supercoiled monomers that topo II treatment is expected to produce. The band assignment is based on the results shown in Figure 1. (C) FACS analysis of DNA content to monitor cell cycle progression during the experiment shown in Figure 4C.

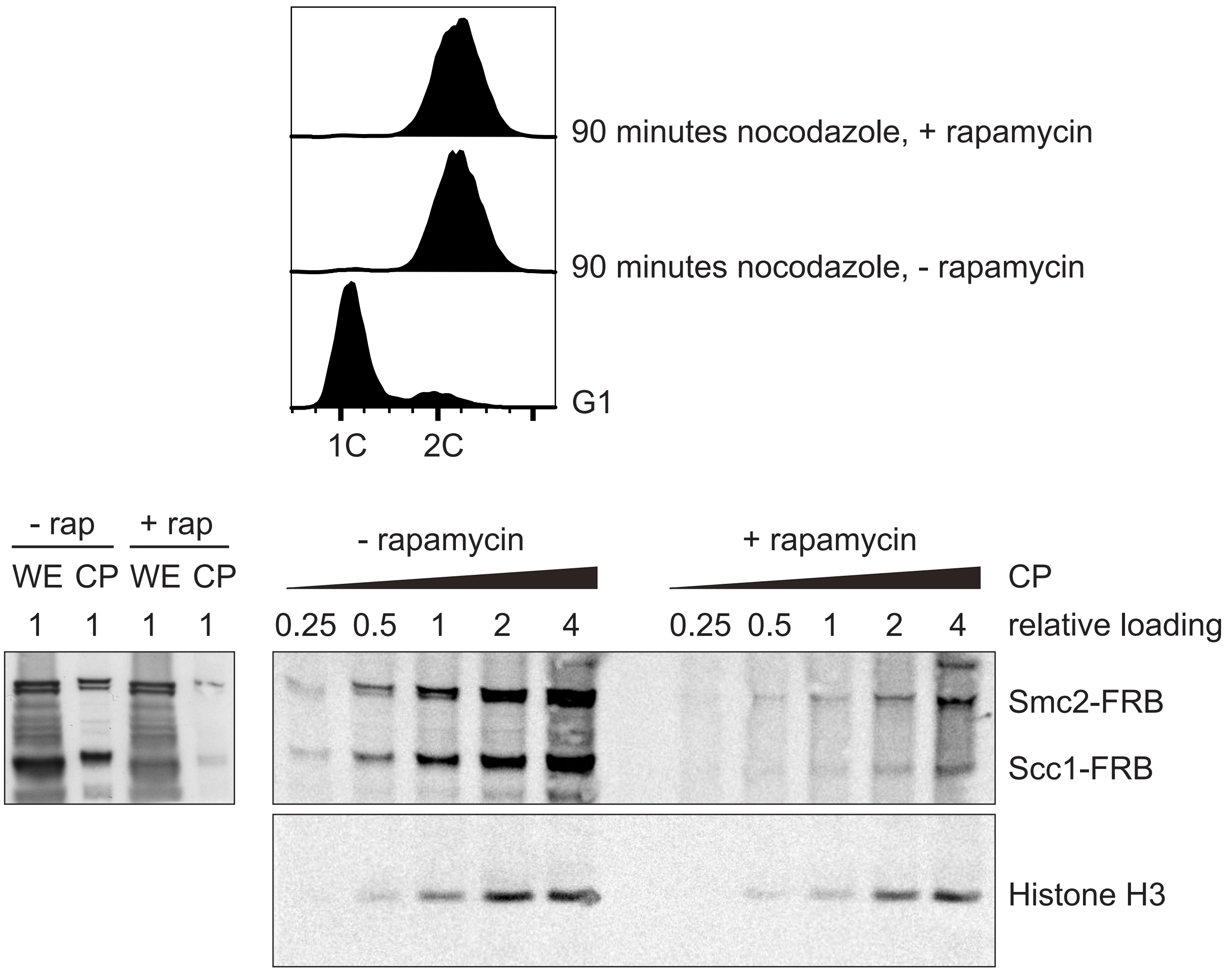

Supplementary Figure S2. Depletion of condensin (Smc2) and cohesin (Scc1) from chromosomes using the anchor-away technique (30). Cells were arrested in G1 using **a**-factor, then the culture was split and released to progress through the cell cycle, here in the presence of nocodazole for subsequent arrest in mitosis (compare e.g. Supplementary Figure S4). Rapamycin was added to half of the culture at the time of release, while the other half received solvent methanol only. 90 minutes after release, virtually all cells had passed through S-phase, as seen by FACS analysis of DNA content. Whole cell extracts (WE) were prepared and fractionated into soluble proteins and chromatin pellets (CP), as previously described (38). Levels of Smc2 and Scc1 in the whole cell extracts and in the chromatin fractions were analyzed by Western blotting using an α-FRB antibody (Alexis Biochemical). An α-histone H3 antibody (Santa Cruz Biotechnology) was used as a loading control for normalization. Western blots were quantitatively analyzed using an ImageQuant LAS 4000 digital imaging system. This revealed that upon rapamycin addition the chromatin-bound levels of Smc2 were reduced to below 20% of the control, while total cellular Smc2 levels remained unchanged. Scc1 levels on chromatin after rapamycin treatment were reduced to less than 10% of the control. The Scc1 concentration in the whole cell extracts was also reduced, probably because Scc1 is less stable when not bound to chromosomes as the consequence of its anchor-away.

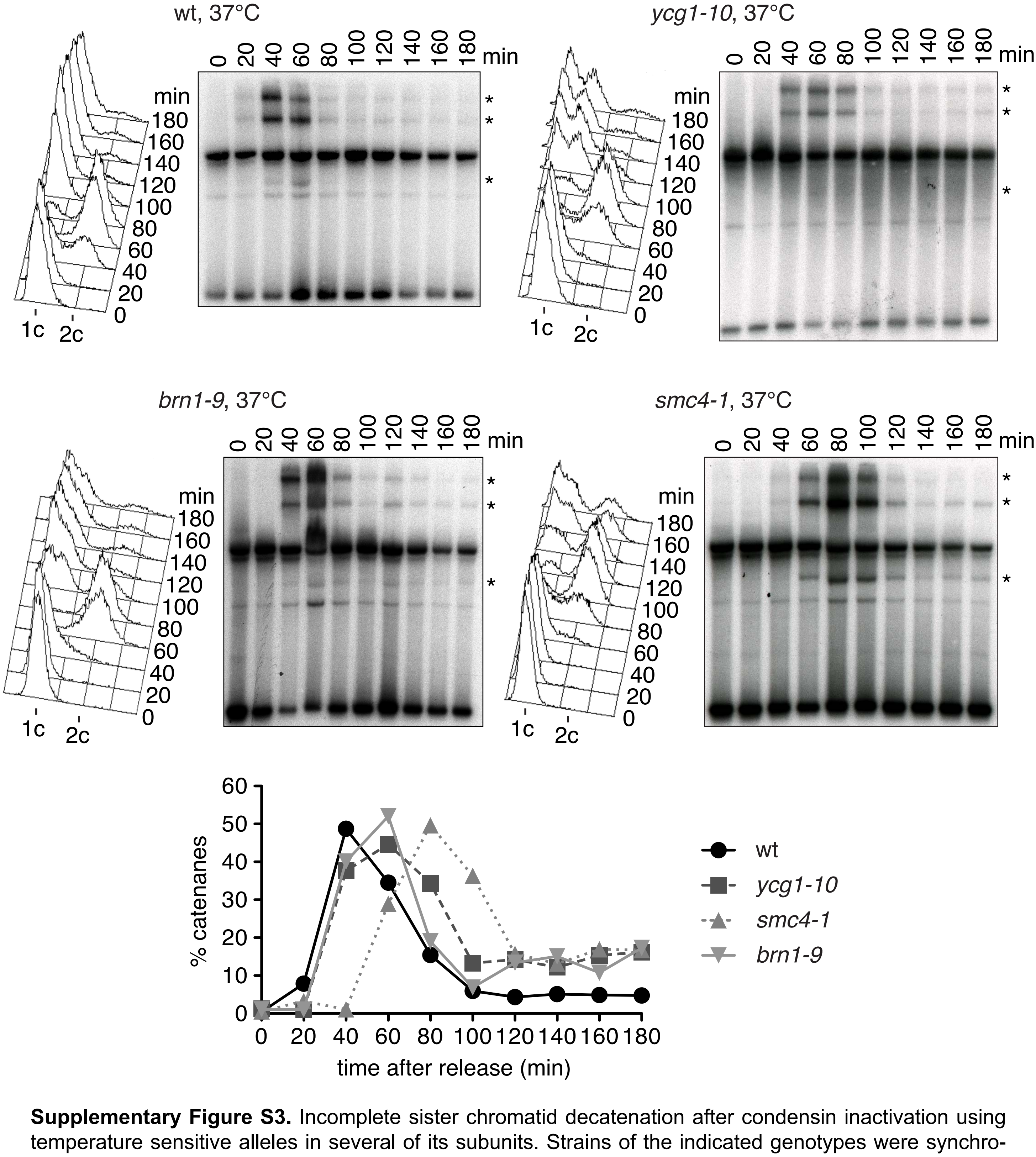

Supplementary Figure S3. Incomplete sister chromatid decatenation after condensin inactivation using temperature sensitive alleles in several of its subunits. Strains of the indicated genotypes were synchronized in G1 and released to progress through the cell cycle at 37°C, a restrictive temperature for the conditional alleles. α-factor was added back to the cultures after release for re-arrest in the next G1. FACS analysis of DNA content is shown together with the Southern blots to visualize the minichromosome pS14-8. Catenanes are highlighted by asterisks (*). Catenane resolution was incomplete in wild type cells at the elevated temperature, making the comparison with the condensin mutants harder. Nevertheless, quantification shows a greater level of persisting catenanes in each of the three mutants, consistent with the results obtained with the condensin anchor-away allele presented in Figure 2.

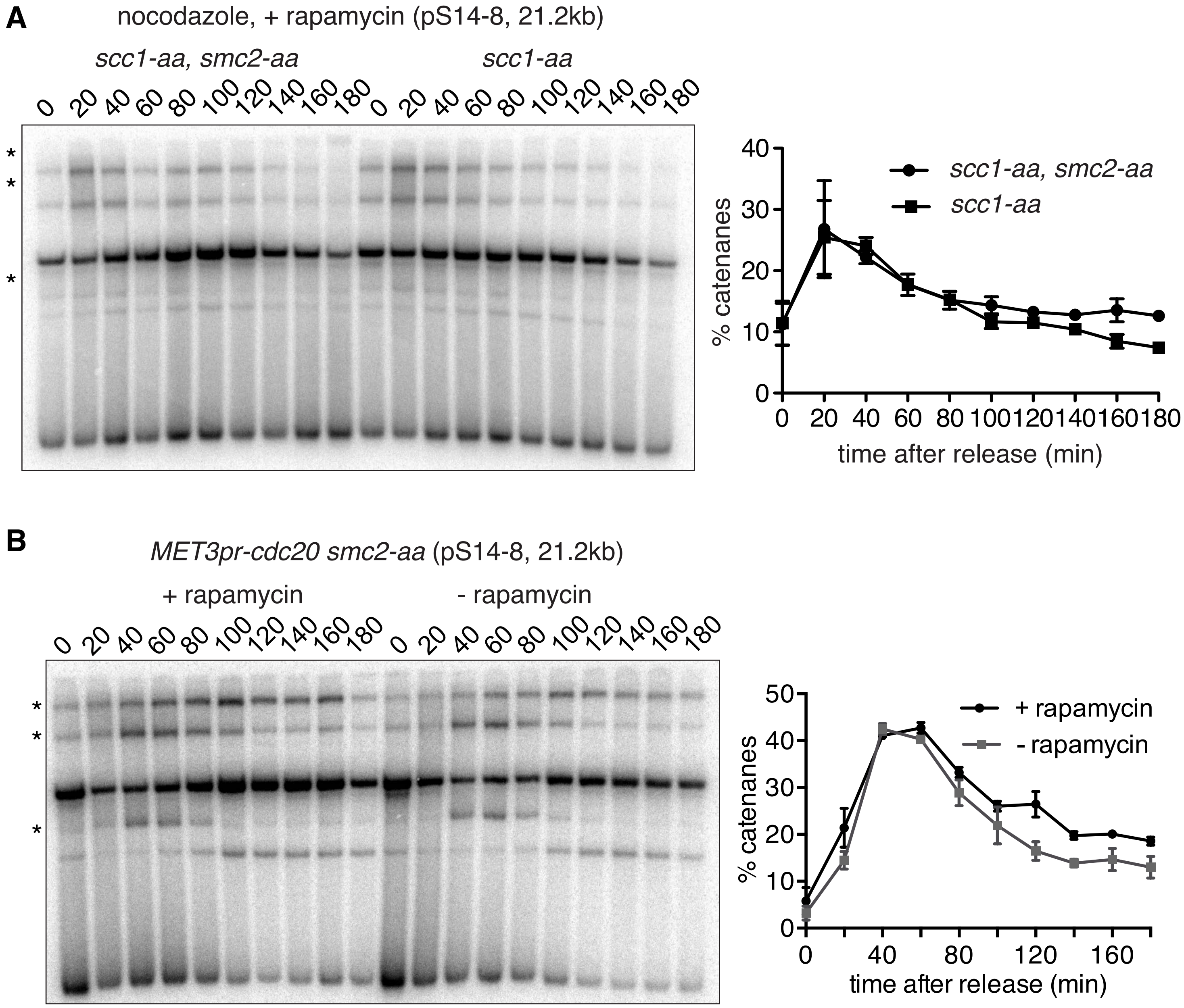

Supplementary Figure S4. Condensin contributes to decatenation independently of chromosome movement. **(A)** Cells of the indicated genotypes were arrested in G1 using **a**-factor and released to progress through the cell cycle in the presence of nocodazole. The percentage of catenanes (indicated on the Southern blots by asterisks) was quantified and plotted over time. The experiment was repeated three times, the mean and standard error are shown. In the absence of cohesin and condensin, a plateau of approximately 15% catenanes is reached, consistent with the level of persistent catenanes observed in condensin-depleted cells under other conditions (compare Figure 2 and Supplementary Figure S3). In the absence of only cohesin, decatenation proceeds further but remains sluggish, probably because sister chromatid separation is blocked by nocodazole. (**B**) *smc2-aa* cells were released from G1 into a mitotic arrest due to Cdc20 depletion in presence or absence of rapamycin to anchor-away condensin. Under these conditions, a mitotic spindle forms and exerts tension on sister chromatids, but their segregation is impeded by cohesin. Catenanes were quantified and plotted over time, the mean and standard error of two independent experiments are shown. In the absence of condensin a greater fraction of catenanes remains unresolved. Taken together, condensin contributes to sister chromatid decatenation also under conditions when chromosomes do not segregate.

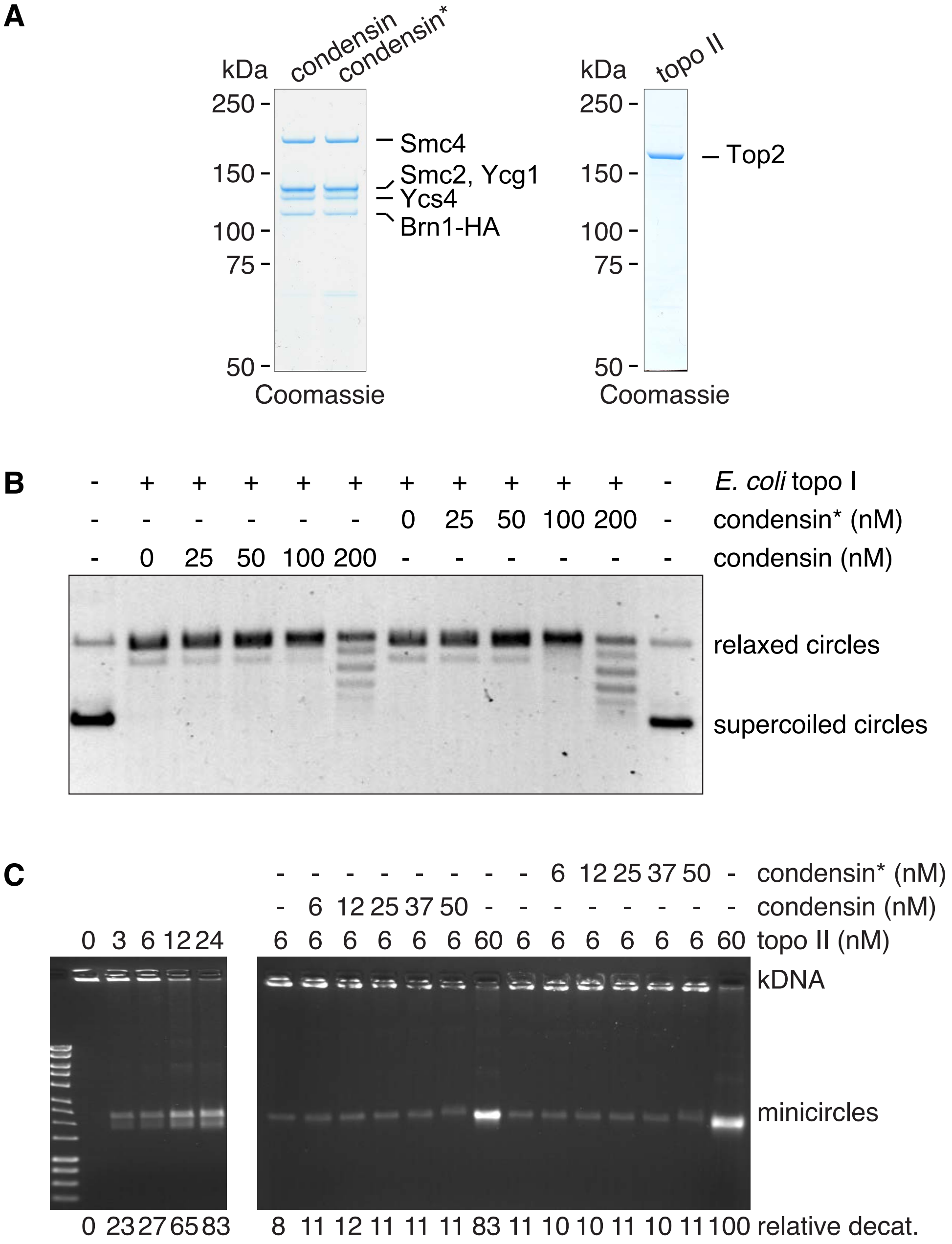

Supplementary Figure S5. Budding yeast condensin does not stimulate *in vitro* kinetoplast (kDNA) decatenation by budding yeast topo II. (**A**) Purification of budding yeast condensin and topo II. Condensin was overexpressed in exponentially growing (condensin) or nocodazole-arrested (condensin*) budding yeast cells as described (39), followed by α-HA affinity purification and HA peptide elution. Topo II was purified as described (40). Purified fractions were analyzed by SDS-PAGE, followed by Coomassie Blue staining. (**B**) Purified condensin is active in DNA supercoiling, a hallmark biochemical activity (41). pBR322 was relaxed by topo I treatment (*E. coli*, New England Biolabs) and purified. 100 ng of relaxed pBR322 (0.9 nM) was incubated with increasing concentrations of condensin or condensin* in the presence of 1 Unit topo I for 1 hour at 30°C. The reaction was stopped by SDS, proteins removed by proteinase K digest and the plasmid topology analyzed by 0.7% agarose/TBE electrophoresis followed by ethidium bromide staining. (**C**) Condensin does not stimulate kDNA decatenation by topo II. 100 ng kDNA was incubated for 20 minutes at 37°C with the indicated concentrations of topo II and condensin or condensin*. kDNA minicircles, released by decatenation, were quantified following ethidium bromide staining. The relative amount of minicircles released is indicated (last lane = 100).

Supplementary References.

38. Liang, C. and Stillman, B. (1997) Persistent initiation of DNA replication and chromatin-bound MCM proteins during the cell cycle in *cdc6* mutants. *Genes Dev.*, **11**, 3375-3386.

39. St-Pierre, J., Douziech, M., Bazile, F., Pascariu, M., Bonneil, E., Sauvé, V., Ratsima, H. and D'Amours, D. (2009) Polo kinase regulates mitotic chromosome condensation by hyperactivation of condensin DNA supercoiling activity. *Mol. Cell*, **34**, 416-426.

40. Worland, S.T. and Wang, J.C. (1989) Inducible overexpression, purification, and active site mapping of DNA topoisomerase II from the yeast *Saccharomyces cerevisiae*. *J. Biol. Chem.*, **264**, 4412-4416.

41. Kimura, K. and Hirano, T. (1997) ATP-Dependent positive supercoiling of DNA by 13S condensin: a biochemical implication for chromosome condensation. *Cell*, **90**, 625-634.
